# The Gut Microbial Architecture of Efficiency Traits in the Domestic Poultry Model Species Japanese Quail (*Coturnix japonica*) Assessed by Mixed Linear Models

**DOI:** 10.1534/g3.120.401424

**Published:** 2020-05-29

**Authors:** Solveig Vollmar, Robin Wellmann, Daniel Borda-Molina, Markus Rodehutscord, Amélia Camarinha-Silva, Jörn Bennewitz

**Affiliations:** Institute of Animal Science, University of Hohenheim, Stuttgart, Germany

**Keywords:** Japanese quail, quantitative traits, microbiability, hologenomic selection

## Abstract

It is well known that mammals and avian gut microbiota compositions are shaped by the host genomes and affect quantitative traits. The microbial architecture describes the impact of the microbiota composition on quantitative trait variation and the number and effect distribution of microbiota features. In the present study the gut microbial architecture of feed-related traits phosphorus and calcium utilization, daily gain, feed intake and feed per gain ratio in the domestic poultry model species Japanese quail were assessed by mixed linear models. The ileum microbiota composition was characterized by 16S rRNA amplicon sequencing techniques of growing individuals. The microbiability of the traits was on a similar level as the narrow sense heritability and was highly significant except for calcium utilization. The animal microbial correlation of the traits was substantial. Microbiome-wide association analyses revealed several traits associated and highly significant microbiota features, both on the bacteria genera as well as on the operational taxonomic unit level. Most features were significant for more than one trait, which explained the high microbial correlations. It can be concluded that the traits are polymicrobial determined with some microbiota features with larger effects and many with small effects. The results are important for the development of hologenomic selection schemes for feed-related traits in avian breeding programs that are targeting the host genome and the metagenome simultaneously.

Livestock microbiota research has received substantial attention in recent years (Estellé 2019). This is driven by the development of cost-effective methods for the characterization of the microbiota composition, *e.g.*, by the 16S rRNA amplicon sequencing approach or by sequencing the entire metagenome. The microbiota composition in the gastrointestinal tract (GIT) is strongly associated with quantitative traits such as growth and feed efficiency traits in pigs (Camarinha-Silva *et al.*, 2017; Maltecca *et al.*, 2019; Quan *et al.*, 2018; Yang *et al.*, 2017), methane emission in ruminants (Difford *et al.*, 2018; Myer 2019; Roehe *et al.*, 2016), and body weight gain and feed per gain ratio in poultry (Meng *et al.*, 2014; Stanley *et al.*, 2012). It is well known that the microbiota itself is shaped by the host genomes and, hence, it can be seen as a mediator between the individual host genome and corresponding quantitative trait records. This enables the development of hologenomic selection schemes that are targeting the host genome and the metagenome simultaneously (Estellé 2019; Weishaar *et al.*, 2020). It was shown that especially for feed-related traits like feed or nutrient efficiency, hologenomic selection is a promising method to alleviate negative side effects of improving these traits on animal health (Weishaar *et al.*, 2020).

Hologenomic selection requires the prediction of quantitative traits with the aid of microbiota composition (Camarinha-Silva *et al.*, 2017; Maltecca *et al.*, 2019; Verschuren *et al.*, 2020) and this benefits from the knowledge of the microbial architecture of quantitative traits. The microbial architecture of a quantitative trait describes the impact of the microbiota composition in a specific GIT section, and the number and effect distribution of microbiota features affecting the trait. This can be assessed with the aid of microbial mixed linear models (Camarinha-Silva *et al.*, 2017; Difford *et al.*, 2018). These models contain a random animal effect with a covariance structure modeled by a microbial relationship matrix M. The elements of M are estimated from the relative microbiota operational taxonomic unit (OTU) abundances shared by pairs of animals. The microbiability (Difford *et al.*, 2018) is the fraction of the phenotypic variance of a trait that can be explained by the microbiota composition. The marginal OTU effects can be obtained from the predicted animal effects. These models can thus be used for a multi-OTU microbiome-wide association study (MWAS), where all OTUs are fitted simultaneously. Expanding these models toward multivariate applications reveal the microbiota-driven trait correlations. Alternatively, single OTUs or bacterial genera can be used one by one in a mixed linear model to test them for trait association. The MWAS approaches can be used to identify the drives for the microbiota trait interrelation (Gilbert *et al.*, 2016).

Japanese quail are well-established model animals in domestic poultry studies because of their short generation interval, small body size, low space requirements, and good comparability to other poultry species (Cheng *et al.*, 2010; Kayang *et al.*, 2004; Mills *et al.*, 1997; Rodehutscord and Dieckmann 2005; Shibusawa *et al.*, 2001; Stock and Bunch 1982). Only a few studies characterizing the GIT microbiota of Japanese quail were conducted (Borda-Molina *et al.*, 2020; Liu *et al.*, 2015; 2018; Wilkinson *et al.*, 2016; 2020). Compared to mammals, the avian GIT is shorter in relation to body size and digesta has a faster passage rate (Wilkinson *et al.*, 2016). While the upper GIT segments (crop, proventriculus and gizzard) are responsible for initial feed hydrolysis, the main nutrient absorption takes place in the small intestine (duodenum, jejunum, and ileum). Thus, the ileum is a suitable location for the microbiota characterization if the interrelation between the microbiota and feed efficiency traits is to be investigated. The paired caeca are particularly important for fermentation and a high microbiota density and diversity is observed in this part of the GIT (Witzig *et al.*, 2015; Yeoman *et al.*, 2012).

Growing Japanese quail were used to study the variability of mineral utilization efficiency, growth, and other efficiency traits by Beck *et al.* (2016). A substantial phenotypic variability of these traits and a significant heritability were reported. Given the importance of the microbiota composition for efficiency traits observed in other species (Maltecca *et al.*, 2019), it can be hypothesized that next to the host genome, feed and nutrient efficiency traits are also affected by the GIT microbiota composition. This is supported by studies on the effect of phosphorus (P) supply on the activity and composition of the microbiota in the ileum and other GIT sections in broiler chickens (Borda-Molina *et al.*, 2016; Ptak *et al.*, 2015; Tilocca *et al.*, 2016; Witzig *et al.*, 2015).

To the best of our knowledge, no studies are published so far analyzing the impact of GIT microbiota on feed-related traits in poultry using microbial mixed linear models and microbiome-wide approaches. The aim of the study was the estimation of microbial parameters for the traits phosphorus utilization (PU), calcium utilization (CaU), feed intake (FI), feed per gain ratio (F:G), and body weight gain (BWG), as well as the application of MWAS on phylum, genera, and OTU level. The interrelation between the traits and the microbiota composition was further assessed with functional predictions.

## Material And Methods

### Experimental design

The experiment was conducted in accordance with the German Animal Welfare Legislation approved by the Animal Welfare Commissioner of the University (approval number S371/13TE) and described in detail by Beck *et al.* (2016). Briefly, a F2 cross of 920 individuals of Japanese quail (*Coturnix japonica*) was established. After plausibility testing, 888 individuals were available for further analyses. Before the quail were individually placed in metabolic units on day five of life, they were housed in groups. After five days of acclimatization to the metabolic units, the performance testing was conducted in a strong growth period between 10^th^ and 15^th^ day of life, and animals were then slaughtered. Slaughtering took place at 12 different days, subsequently denoted as test-days. At slaughter the ileum was longitudinally opened and digesta was collected and stored in RNAlater at -80° until further analysis. The animals were provided with a low-phosphorus but otherwise nutrient-adequate diet. Bodyweight gain (BWG) was calculated as the difference of the body weight at day 10 and day 15. Feed per gain ratio (F:G) was calculated as feed intake (FI) within these 5 days divided by BWG. Phosphorus utilization (PU) and Calcium utilization (CaU) were calculated as the difference between total intake and total excretion of the respective element. Summary statistics are shown in Table 1. Genetic parameters (heritability and genetic correlations) were estimated using mixed linear models and are reported by Beck *et al.* (2016).

**Table 1:** Overview of phenotypic traits. Traits, trait abbreviations, mean, minimum (min), maximum (max) and standard deviation (SD) of the observed traits of the Japanese quail animals

Trait*^a^*	abbreviation	unit	min	mean	max	SD
P utilization	PU	%	21.490	71.399	87.430	7.998
Feed intake	FI	g	16.110	42.630	62.350	7.120
Bodyweight gain	BWG	g	5.800	24.491	37.850	5.032
Feed per gain ratio	F:G	g/g	1.210	1.782	3.920	0.303
Ca utilization	CaU	%	19.420	60.554	84.310	10.018

a From day 10 to 15 of life.

### Ileum microbiota characterization

Ileum microbial composition was obtained from a previous study (Borda-Molina *et al.*, 2020). Briefly, ileum digesta samples of 760 quails were sequenced using 250bp paired-end sequencing chemistry on an Ilumina MiSeq platform (128 samples did not pass the quality filter of the sequences and were subsequently discarded). Demultiplexing and trimming of sequencing reads were done by using the default parameters from QIIME v1.9.1 pipeline (Caporaso *et al.*, 2010), and it followed a subsampled open-reference OTU (operational taxonomic units) calling approach of the pipeline, with a maximum sequence length of 360 bp. The reads were merged into one fasta file and aligned using the SILVA Database (Release 132) (Quast *et al.*, 2013). We used this database, because of its data are quality checked and includes more updated information. Chimeras were identified and removed using usearch (Edgar *et al.*, 2011). Sequence reads can be accessed under the accession number PREJB37544. Sequences were clustered into operational taxonomic units (OTU) at >97% similarity and were taxonomically assigned to the closest species. OTUs were standardized by total. For further analyses, OTUs with an abundance lower than 0.0001% were removed and only phyla and genera with an average abundance higher than 0.5% are displayed in the results.

Functional predictions were carried out with the R package Tax4Fun2 (Wemheuer *et al.*, 2020), which relied on the SILVA database (Yilmaz *et al.*, 2014) and used the KEGG hierarchy for the assignations (Kanehisa *et al.*, 2016). Silva database can provide more accurate information because it is regularly updated and maintained, and taxonomic assignations are manually curated (Balvočiūtė and Huson 2017). The biom table to assign this functionality was obtained from qiime pipeline (McDonald *et al.*, 2012). Genomes from 16S rRNA gene sequences identified in this study were downloaded from the NCBI database (https://www.ncbi.nlm.nih.gov/home/genomes/) in order to produce the most accurate database. Functional predictions were correlated with the quantitative traits.

### Statistical analyses

#### Microbial linear mixed model:

All statistical analyses were performed in R Studio (Version 3.5.2). The following microbial mixed linear model was fitted within ASReml R (Version 3.0) (Butler *et al.*, 2009) to determine the microbial variance components:

$$y=µ\;1+Z_{td}td+m+e,$$ (1)

where y is the vector with trait records (the considered traits were PU, BWG, FI, F:G, and CaU), µ is the trait mean and 1 is the vector of ones, vector $td∼N\left(0,I\sigma_{td}^{2}\right)$ is the vector of random test day (*i.e.*, the effect of the day at slaughter) effects with variance $\sigma_{td}^{2}$ and design matrix Z, and vector $e∼N\left(0,I\sigma_{e}^{2}\right)$ contains the random residuals with variance $\sigma_{e}^{2}$. Vector m contains the random microbiota animal effects with distribution $m∼\;N\left(0,\;M\;\sigma_{m}^{2}\right)$ and microbial variance $\sigma_{m}^{2}$. The microbial relationship matrix M was calculated as $M=\frac{XX^{T}}{N}$, where *N* is the number of OTUs and *X* is a n×N matrix, where n is the number of animals. Matrix *X* contains the standardized and log-transformed abundances of the OTUs (Camarinha-Silva *et al.*, 2017). The model was applied in an univariate setting for the estimation of microbiability ($m^{2}$) as $m^{2}=\;\frac{\sigma_{m}^{2}\;}{\sigma_{p}^{2}\;}$, with $\sigma_{p}^{2}=\sigma_{m}^{2}+\;\sigma_{td}^{2}+\;\sigma_{e}^{2}$. The significance of microbiability was tested by conducting a likelihood-ratio test on the random animal effects. The test statistic was calculated as $D=2\left[\log\left(L_{2}\right)-\log\left(L_{1}\right)\right]$, with $L_{2}$ being the likelihood of the full model and $L_{1}$ of the reduced model, *i.e.*, model (1) without the random microbiota animal effect. The test statistic *D* under the null-hypothesis was chi-squared distributed with one degree of freedom. Next to the microbiability, the microbiota correlation between quantitative traits was of interest. For this purpose, model (1) was extended toward bivariate applications. The covariance matrix of the random microbiota animal effects became $Var\left[\begin{array}{c} m_{1}\\ m_{2} \end{array}\right]=M⊗\left[\begin{array}{cc} \sigma_{m1}^{2} & \sigma_{m1,m2}\\ \sigma_{m1,m2} & \sigma_{m2}^{2} \end{array}\right]$, with $\sigma_{m1,m2}$ being the covariance of the animal microbiota effects on trait 1 and 2. From the solutions of this bivariate model the animal microbiota correlations were estimated as$\;r_{m1,m2}={\hat{\sigma }}_{m1,m2}/({\hat{\sigma }}_{m1}*{\hat{\sigma }}_{m2})$. The significance of the correlation was tested by a likelihood ratio test as described above, with $L_{2}$ being the likelihood of the full bivariate model and $L_{1}$ of the corresponding bivariate model but with the covariance fixed at zero. In addition, phenotypic correlations between the raw trait records were calculated.

#### Microbiome-wide association analyses, MWAS:

MWAS were conducted using two different approaches. The first approach was applied to bacterial genus level. A second filter step was applied at a minimum of 0.5% mean abundance of a bacterial genus. This reduced the number of genera down to 74, which were subject to the association analysis using the following mixed linear model

$$y=Xb+Z_{td}td+a+e,$$ (2)

where b is a vector with fixed effects containing the trait mean and the bacterial genus to be tested. The vector a contained the random animal effect with distribution $a∼\;N\left(0,\;A\sigma_{a}^{2}\right)$, where *A* is the pedigree-based relationship matrix and $\sigma_{a}^{2}$ the additive genetic variance (Lynch and Walsh 1998). The effect of the bacterial genera was modeled as a covariate, *i.e.*, the observation of an individual was regressed on the abundance of the bacteria genera. The regression coefficient was tested for significance using an F-Test. This model was applied for each of the 74 genera and each trait separately. The nominal p-values were corrected for multiple comparisons using the Bonferroni correction method. The correction was applied within each trait. To judge how many false-positive results were among the significant associations we calculated the false-discovery rate (FDR) (Benjamini and Hochberg 1995) using the software QVALUE (Storey and Tibshirani 2003). The FDR q-value of the significant bacterial genera with the lowest test statistic provided an estimate of the proportion of false-positive results among the significant associations.

The same approach was applied on the phylum level, with the four most abundant phyla (mean abundance > 0.5%) being tested. Because multiple testing is not a serious issue here, the nominal p-values were not corrected.

The second MWAS approach was applied at the multi-OTU level. We used model (1) for predicting the animal microbiota effects and obtained OTU effects by back-solving the effects as

$$\hat{u}=\frac{X^{’}M^{-1}\hat{m}}{N},$$ (3)

where $\hat{u}$ is the vector with estimated OTU effects, matrix *X* is as defined above, *N* is the number of OTUs, $M^{-1}$ is the inverted microbial relationship matrix, and $\hat{m}$ is the vector with estimated animal microbiota effects (obtained from model (1)). Because all OTU effects were estimated simultaneously, they can be interpreted as marginal effects, *i.e.*, the effect of each OTU is corrected for the effects of all other OTUs. We examined those OTU whose absolute trait association effect exceeded 0.25$\;\sigma_{m}$ more closely.

### Data availability

All data generated and analyzed during this study were fully uploaded to the database of the journal. Supplemental material available at figshare: https://doi.org/10.25387/g3.12123606.

## Results

### Ileum microbiota community and functional predictions

The amplicon sequences were classified into 1188 OTUs belonging to 7 microbial phyla (Table 2). Most abundant bacterial groups at phylum level included *Firmicutes* (mean abundance in percentages 83.25), followed by *Proteobacteria* (mean abundance 14.29), *Actinobacteria* (mean abundance 1.65), and *Bacteroidetes* (mean abundance 0.70). The remaining phyla were identified as *Epsilonbacteraeota*, *Tenericutes*, and *others*. The most abundant genera were *Candidatus Arthromitus* (mean abundance 29.64), *Clostridium sensu stricto* (mean abundance 14.11), *Enterococcus* (mean abundance 3.75), *Escherichia-Shigella* (mean abundance 14.17), *Lactobacillus* (mean abundance 24.33) and *Streptococcus* (mean abundance 8.25). They account for 96% of the total community. Further details regarding the microbiota characteristic are presented in (Borda-Molina *et al.*, 2020).

**Table 2:** Sample distribution at phylum level. Relative abundances at the phylum level with their minimal (min), mean, maximum (max) values, and standard deviation (SD)

Phylum	Relative abundances			SD
	min	mean	max	
*Actinobacteria*	0.002	1.652	39.921	3.424
*Bacteroidetes*	<0.001	0.698	41.246	2.947
*Epsilonbacteraeota*	<0.001	<0.001	0.044	0.003
*Firmicutes*	16.393	83.249	99.875	12.718
*others*	<0.001	0.104	1.206	0.126
*Proteobacteria*	0.028	14.295	81.490	12.066
*Tenericutes*	<0.001	0.001	0.194	0.012

**Figure 1:**
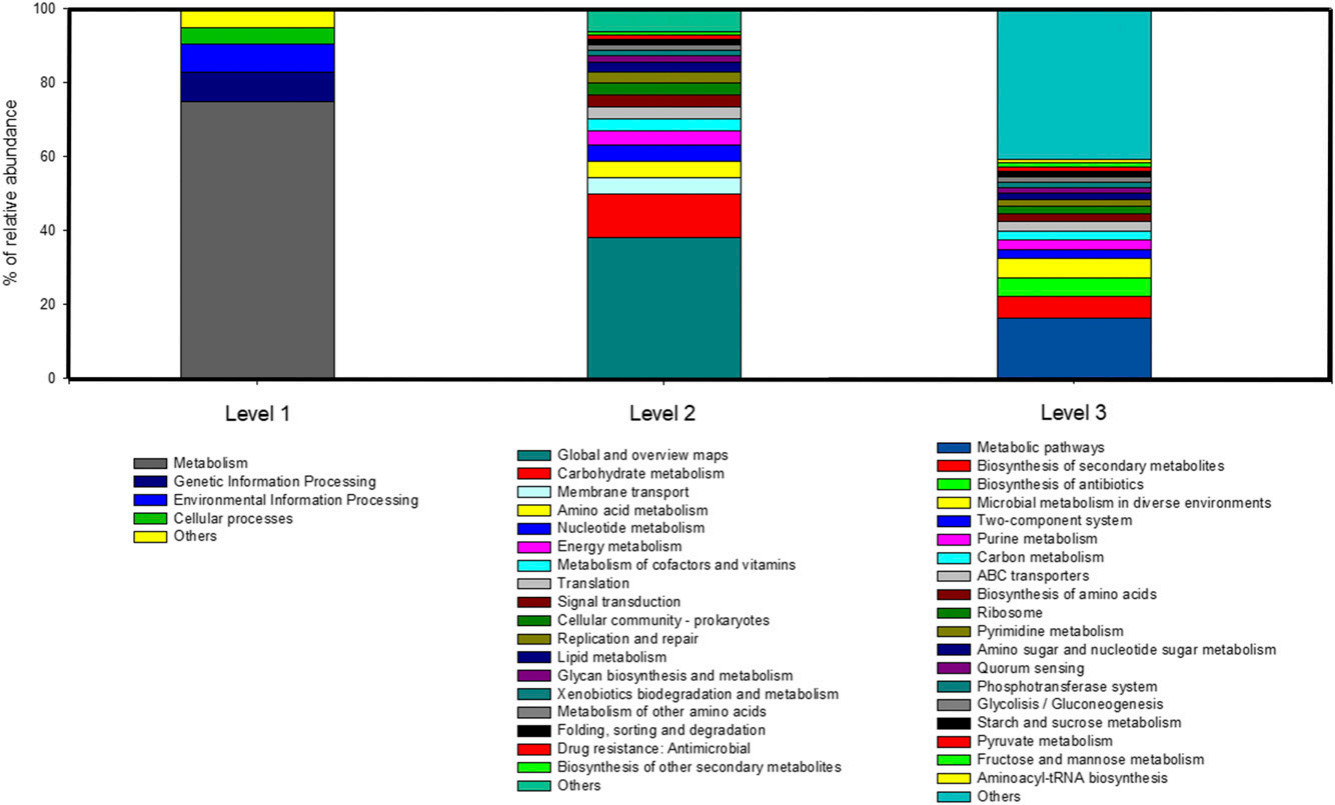
Functional predictions of different levels of classification. Bar plot for the percentage of relative abundances (y-axis) of the predicted functions (x-axis) at the three levels of classification based on KEGG database.

The results from the functional predictions are shown in Figure 1 for three classification levels. At the broadest level of classification (level 1), the main activities were carried out for metabolism, followed by genetic information processing, and environmental information processing. In the next classification level (level 2 in Figure 1) the most abundant activities comprised carbohydrate metabolism, amino acid metabolism and nucleotide metabolism, energy metabolism, metabolism of cofactors and vitamins, and lipid metabolism (Figure 1 and Table S1).

From 352 predicted functions at the third level, a number of significant correlations with the quantitative traits were identified (Table S2). To summarize, for PU a total of 17 positive correlations with functions related to metabolism and environmental information processing were found. CaU was positively correlated with 30 functions belonging mainly to metabolism and five negative interactions. BWG showed 48 positive interactions with metabolism and 18 negative interactions. F:G registered 67 positive and 35 negative interactions. The highest number of correlations were registered with feed intake where 112 were positive and 60 were negative (Table S1). Thus, all the traits evaluated mainly interact with metabolic classified predicted functions.

### Microbial parameters

The estimated microbiabilities (results from model 1) were low for CaU and FI, and moderate for PU, BWG, and F:G (Table 3). They were highly significant with small standard errors, except for CaU (*P* = 0.23). Therefore, no further microbial analyses were conducted for CaU. The test-day variance component (Table 3) was small for all traits, except for FI.

**Table 3:** Results from the microbial linear mixed model (model 1), with microbial variance ($\sigma_{m}^{2}$), test-day variance ($\sigma_{td}^{2}$), residual variance ($\sigma_{e}^{2}$), and microbiability (m^2^) with p values (standard errors are in in parenthesis)

Trait*^a^*	$\sigma_{m}^{2}$ (SE)	$\sigma_{\mathrm{td}}^{2}$ (SE)	$\sigma_{e}^{2}$ (SE)	$m^{2}$ (SE)	p value
PU	9.083 (3.210)	1.278 (0.997)	50.043 (3.228)	0.150 (0.050)	<0.001
FI	4.603 (1.852)	9.918 (4.618)	35.152 (2.169)	0.093 (0.037)	<0.001
BWG	4.302 (1.242)	1.504 (0.842)	17.973 (1.160)	0.181 (0.048)	<0.001
F:G	0.023 (0.005)	0.001 (0.001)	0.061 (0.004)	0.269 (0.051)	<0.001
CaU	4.463 (3.771)	5.846 (3.278)	91.457 (5.526)	0.044 (0.037)	0.235

a For trait abbreviations see Table 1.

The animal microbial correlations (results from the bivariate extensions of model 1, Table 4) were substantial. They were close to one for BWG-F:G and above 0.9 for PU-FI, and FI-BWG. They were highly significant and the standard errors were small in relation to the estimates. The microbial correlation coefficients were much larger than the phenotypic correlations, but the directions were the same.

**Table 4:** Phenotypic *vs.* animal microbial correlations. Phenotypic correlations ($r_{pearson}$) and results from the bivariate microbial linear mixed model (bivariate extensions of model 1), with microbial covariance ($\sigma_{m1,m2}$), and microbial correlation ($r_{m1,m2}$) with p values (standard errors are in in parenthesis)

Traits*^a^*	Phenotypic correlation		Animal microbial correlation		
	$r_{pearson}$	p value	$\sigma_{m1,m2}$ (SE)	$r_{m1,m2}$ (SE)	p value
PU – FI	0.561	<0.001	5.695 (2.085)	0.905 (0.102)	<0.001
PU – BWG	0.581	<0.001	4.671 (1.637)	0.791 (0.116)	<0.001
PU – F:G	−0.387	<0.001	−0.310 (0.097)	−0.738 (0.134)	<0.001
FI – BWG	0.849	<0.001	3.743 (1.346)	0.902 (0.059)	<0.001
FI – F:G	−0.213	<0.001	−0.282 (0.076)	−0.876 (0.117)	<0.001
BWG – F:G	−0.645	<0.001	−0.302 (0.072)	−0.982 (0.028)	<0.001

a For trait abbreviations see Table 1.

### Microbiome-wide association analyses

The results of the single-feature MWAS (model 2) for the four most abundant phyla revealed only weak significant associations for *Firmicutes* and *Proteobacteria* with PU. A higher abundance of *Firmicutes* increased (*P* nominal = 0.016) and a higher abundance of *Proteobacteria* decreased PU (*P* nominal = 0.048) (not shown elsewhere).

All genera and OTU effects are reported in units of $\sigma_{m}$. The significant associations (*P* nominal < 0.05) on the genus level are shown in Table 5. The number of microbiome-wide significant associations (p adjusted < 0.05) were 2 (3, 5, 6) for PU (FI, BWG, F:G, respectively). Remarkably, some genera showed highly significant associations for multiple traits. These were *Kurthia* (all four traits), *Candidatus Arthromitus* (PU, BWG, and FI), *Leuconostoc* (PU and BWG), *Enterococcus* and *Rothia* (both for BWG and F:G). All four PU significant genera were also significant for FI and BWG. The sign of some effects were in agreement with the signs of the microbial correlation coefficients (Table 4). The highest number of significant associations among the traits was found for F:G.

**Table 5:** Results from the MWAS conducted with model (2) at the genus level (n = 74) with nominal p and adjusted p values, FDR q values, effect estimates $\hat{b}$ (in units $\sigma_{m}$, standard errors are in parenthesis)

Trait*^a^*	Genus	P value	FDR q-value	p adjusted	$\hat{b}$ (SE)
PU	*Candidatus Arthromitus*	<0.001	<0.001	<0.001	0.024 (0.005)
	*Kurthia*	<0.001	0.011	0.022	−1.133 (0.312)
	*Leuconostoc*	0.005	0.089	0.291	1.083 (0.381)
	*Bacillus*	0.005	0.089	0.301	1.677 (0.593)
FI	*Candidatus Arthromitus*	<0.001	<0.001	<0.001	0.033 (0.006)
	*Kurthia*	<0.001	0.009	0.019	−1.329 (0.362)
	*Leuconostoc*	0.001	0.015	0.044	1.545 (0.449)
	*Enterococcus*	0.001	0.018	0.068	−0.040 (0.012)
	*Bacillus*	0.008	0.104	0.467	1.852 (0.702)
	*Streptococcus*	0.010	0.105	0.521	−0.018 (0.007)
BWG	*Candidatus Arthromitus*	<0.001	<0.001	<0.001	0.026 (0.005)
	*Enterococcus*	<0.001	<0.001	0.001	−0.040 (0.009)
	*Kurthia*	<0.001	0.001	0.002	−1.176 (0.281)
	*Leuconostoc*	<0.001	0.006	0.025	1.252 (0.348)
	*Rothia*	<0.001	0.006	0.028	−0.666 (0.186)
	*Streptococcus*	0.001	0.008	0.059	−0.018 (0.005)
	*Macrococcus*	0.001	0.008	0.064	−0.311 (0.093)
	*Aerococcus*	0.002	0.016	0.137	−0.158 (0.051)
	Unclassified Clostridiaceae1	0.002	0.016	0.145	2.266 (0.734)
	*Clostridium sensu stricto*	0.015	0.102	0.675	0.013 (0.005)
	*Propionibacterium*	0.023	0.142	0.822	2.987 (1.312)
	*Clostridium XlVa*	0.026	0.146	0.853	−1.022 (0.457)
	*Bacillus*	0.028	0.146	0.874	1.204 (0.546)
	*Erysipelotrichaceae incertae sedis*	0.030	0.147	0.893	−3.053 (1.402)
F:G	*Aerococcus*	<0.001	<0.001	<0.001	0.211 (0.042)
	*Kurthia*	<0.001	<0.001	<0.001	1.147 (0.233)
	*Staphylococcus*	<0.001	<0.001	0.001	0.317 (0.073)
	*Enterococcus*	<0.001	0.002	0.006	0.033 (0.008)
	*Rothia*	<0.001	0.002	0.009	0.600 (0.155)
	*Macrococcus*	0.001	0.009	0.050	0.264 (0.077)
	Unclassified Ruminococcaceae	0.001	0.009	0.061	0.613 (0.183)
	*Cutibacterium*	0.003	0.021	0.170	0.620 (0.205)
	*Subdoligranulum*	0.003	0.021	0.174	0.745 (0.247)
	*Candidatus Arthromitus*	0.004	0.026	0.230	−0.013 (0.004)
	Erysipelotrichaceae *incertae sedis*	0.004	0.028	0.265	3.350 (1.166)
	Unclassified Lachnospiraceae	0.005	0.028	0.290	0.171 (0.060)
	Lachnospiraceae *incertae sedis*	0.008	0.044	0.440	1.583 (0.594)
	*Clostridium sensu stricto*	0.010	0.055	0.542	−0.013 (0.004)
	*Streptococcus*	0.021	0.106	0.798	0.013 (0.004)
	*Clostridium XlVa*	0.027	0.120	0.867	0.844 (0.380)
	*Sellimonas*	0.028	0.120	0.875	1.385 (0.629)

a For trait abbreviations see Table 1.

**Figure 2:**
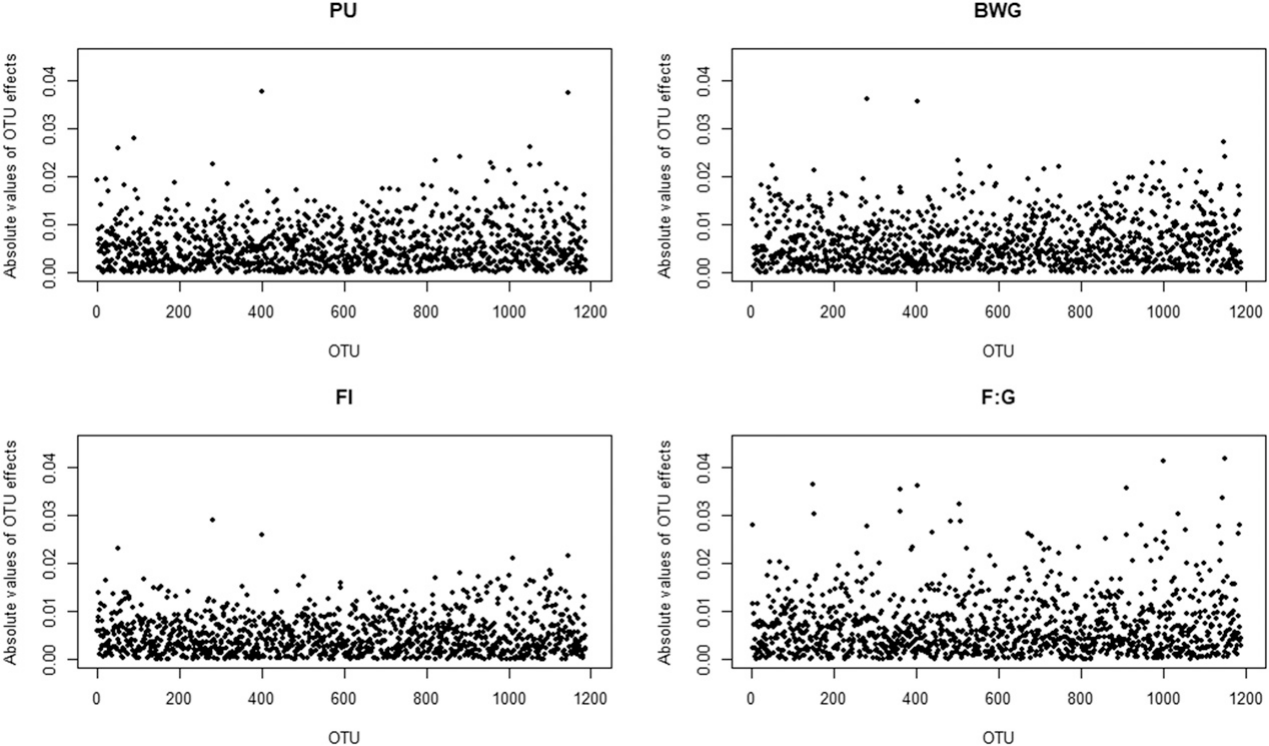
Manhattan plot of results from the microbiome-wide association study conducted with model (3) for P utilization (PU), feed intake (FI), body weight gain (BWG), and feed per gain (F:G). Each dot represents absolute marginal OTU effect in units of $\sigma_{m}$ and the corresponding OTU number.

The results from the multi-OTU MWAS (model 3) are shown as Manhattan plots of marginal OTU effects in Figure 2. Several OTUs with large marginal effects (≥ 0.025$\sigma_{m}$) were mapped for all traits and are listed in Table 6 along with their taxonomic classifications. Among the traits, most large effect OTUs were mapped for F:G. Some large OTU affected several traits. The OTU402 showed a large effect for all four traits, OTU281 for FI, BWG, and F:G, and OTU1146 for PU and BWG. The OTU1053 affected both, PU and F:G.

**Table 6:** Results from the MWAS conducted with model (3) with marginal absolute OTU effect estimates $\hat{b}$ (in units $\sigma_{m}$, standard errors are in parenthesis), and taxonomic classification. Results with $\hat{b}$ ≥ 0.025 $\sigma_{m}$ are shown

Trait*^a^*	OTU	$\hat{\text{b}}$	Phylum	Class	Order	Family	Genus
PU	OTU50	0.026	*Firmicutes*	*Bacilli*	*Bacillales*	*Paenibacillaceae*	*Paenibacillus*
	OTU1053	0.026	*Proteobacteria*	*Gammaproteobacteria*	*Enterobacteriales*	*Enterobacteriaceae*	*Escherichia-Shigella*
	OTU90	0.028	*Proteobacteria*	*Gammaproteobacteria*	*Enterobacteriales*	*Enterobacteriaceae*	*Escherichia-Shigella*
	OTU1146	0.037	*Proteobacteria*	*Gammaproteobacteria*	*Enterobacteriales*	*Enterobacteriaceae*	*Escherichia-Shigella*
	OTU402	0.038	*Firmicutes*	*Bacilli*	*Bacillales*	*Paenibacillaceae*	*Paenibacillus*
FI	OTU402	0.026	*Firmicutes*	*Bacilli*	*Bacillales*	*Paenibacillaceae*	*Paenibacillus*
	OTU281	0.029	*Firmicutes*	*Clostridia*	*Clostridiales*	*Ruminococcaceae*	*Unclassified_Ruminococcaceae*
BWG	OTU1146	0.027	*Proteobacteria*	*Gammaproteobacteria*	*Enterobacteriales*	*Enterobacteriaceae*	*Escherichia-Shigella*
	OTU402	0.036	*Firmicutes*	*Bacilli*	*Bacillales*	*Paenibacillaceae*	*Paenibacillus*
	OTU281	0.036	*Firmicutes*	*Clostridia*	*Clostridiales*	*Ruminococcaceae*	*Unclassified_Ruminococcaceae*
F:G	OTU982	0.025	*Actinobacteria*	*Actinobacteria*	*Micrococcales*	*Dermabacteraceae*	*Brachybacterium*
	OTU858	0.025	*Firmicutes*	*Clostridia*	*Clostridiales*	*Clostridiaceae 1*	*Candidatus Arthromitus*
	OTU681	0.026	*Firmicutes*	*Clostridia*	*Clostridiales*	*Clostridiaceae 1*	*Candidatus Arthromitus*
	OTU909	0.026	*Firmicutes*	*Clostridia*	*Clostridiales*	*Ruminococcaceae*	*Unclassified_Ruminococcaceae*
	OTU1183	0.026	*Proteobacteria*	*Gammaproteobacteria*	*Enterobacteriales*	*Enterobacteriaceae*	*Escherichia-Shigella*
	OTU672	0.026	*Actinobacteria*	*Actinobacteria*	*Micrococcales*	*Microbacteriaceae*	*Leucobacter*
	OTU1002	0.026	*Firmicutes*	*Clostridia*	*Clostridiales*	*Lachnospiraceae*	*Tyzzerella*
	OTU437	0.027	*Bacteroidetes*	*Bacteroidia*	*Bacteroidales*	*Bacteroidaceae*	*Bacteroides*
	OTU1053	0.027	*Proteobacteria*	*Gammaproteobacteria*	*Enterobacteriales*	*Enterobacteriaceae*	*Escherichia-Shigella*
	OTU281	0.028	*Firmicutes*	*Clostridia*	*Clostridiales*	*Ruminococcaceae*	*Unclassified_Ruminococcaceae*
	OTU1134	0.028	*Firmicutes*	*Clostridia*	*Clostridiales*	*Ruminococcaceae*	*Unclassified_Ruminococcaceae*
	OTU1	0.028	*Firmicutes*	*Clostridia*	*Clostridiales*	*Lachnospiraceae*	*Ruminococcus2*
	OTU1186	0.028	*Firmicutes*	*Bacilli*	*Bacillales*	*Bacillaceae*	*Bacillus*
	OTU947	0.028	*Firmicutes*	*Bacilli*	*Lactobacillales*	*Lactobacillaceae*	*Lactobacillus*
	OTU483	0.029	*Proteobacteria*	*Gammaproteobacteria*	*Enterobacteriales*	*Enterobacteriaceae*	*Escherichia-Shigella*
	OTU507	0.029	*Proteobacteria*	*Gammaproteobacteria*	*Enterobacteriales*	*Enterobacteriaceae*	*Escherichia-Shigella*
	OTU150	0.030	*Firmicutes*	*Clostridia*	*Clostridiales*	*Ruminococcaceae*	*Unclassified_Ruminococcaceae*
	OTU1037	0.030	*Firmicutes*	*Bacilli*	*Lactobacillales*	*Enterococcaceae*	*Enterococcus*
	OTU359	0.031	*Proteobacteria*	*Gammaproteobacteria*	*Pseudomonadales*	*Moraxellaceae*	*Psychrobacter*
	OTU504	0.032	*Firmicutes*	*Bacilli*	*Bacillales*	*Staphylococcaceae*	*Staphylococcus*
	OTU1143	0.034	*Firmicutes*	*Clostridia*	*Clostridiales*	*Lachnospiraceae*	*Roseburia*
	OTU361	0.035	*Firmicutes*	*Clostridia*	*Clostridiales*	*Lachnospiraceae*	*Lachnospiraceae_incertae_sedis*
	OTU910	0.036	*Firmicutes*	*Clostridia*	*Clostridiales*	*Ruminococcaceae*	*Unclassified_Ruminococcaceae*
	OTU402	0.036	*Firmicutes*	*Bacilli*	*Bacillales*	*Paenibacillaceae*	*Paenibacillus*
	OTU149	0.037	*Firmicutes*	*Clostridia*	*Clostridiales*	*Lachnospiraceae*	*Blautia*
	OTU1001	0.041	*Firmicutes*	*Bacilli*	*Lactobacillales*	*Enterococcaceae*	*Enterococcus*
	OTU1148	0.042	*Firmicutes*	*Clostridia*	*Clostridiales*	*Clostridiaceae 1*	*Unclassified_Clostridiaceae1*

a For trait abbreviations see Table.

## Discussion

This study analyzed the effect of the ileum microbiota composition on multiple quantitative traits with microbial mixed linear models. The results from functional predictions (Figure 1, Table S1, and Table S2) revealed that the ileum of quails is a highly metabolic active microbial environment. The $m^{2}$ estimates (Table 3) revealed a substantial impact of the microbiota composition on F:G and also on BWG and PU, which was also found with the functional predictions (Table S2). The $m^{2}$ estimates were on a similar level as the narrow sense heritability estimates for these traits (Beck *et al.*, 2016). Interestingly, the estimated animal microbiota correlations $r_{m1,m2}$ between traits were markedly high (Table 4), which is due to linkages between the traits, *i.e.*, they were all P- related. It is known from other monogastric species that feed-related traits are affected by the GIT microbiota composition (Maltecca *et al.*, 2020). However, the animal microbiota correlations $r_{m1,m2}$ were larger than the phenotypic correlations (Table 4) and the genetic correlations (Beck *et al.*, 2016). This points to the same underlying microbiota fractions affecting this class of traits. This can also be deduced from the MWAS results (Table 6), where most genera affected more than one trait. Some genera showed substantial effects with up to two or even three units of $\sigma_{m}$, *e.g.*, for BWG (Table 6), even though these estimates may be biased due to multiple testing in the MWAS.

The results from the OTU level MWAS revealed some outliers with marginal effects > 0.025$\sigma_{m}$, with many OTUs affecting more than one trait (Figure 2 and Table 6). However, no substantial peaked OTU could be identified. It might be that the large genera effect obtained from model (2) were dissected down to multiple marginal OTU effects underlying each genus. *Firmicutes* and *Proteobacteria* were also one of the most abundant phyla in other studies (Kumar *et al.*, 2018; Liu *et al.*, 2018; Shah *et al.*, 2019; Su *et al.*, 2014; Wilkinson *et al.*, 2016). From these two phyla, four OTUs were associated with several traits (Table 6). Both OTUs of the phylum *Proteobacteria* belong to the *Escherichia-Shigella* genus, which is known as enteropathogenic microorganism. Both OTUs had negative effects on BWG and PU, while a positive effect was estimated for F:G. In broilers, abundance of *Escherichia-Shigella* in crop, ileum, and caeca samples was negatively correlated with performance traits (Fonseca *et al.*, 2010; Rubio *et al.*, 2015), which is consistent with our estimates for BWG and PU. One common colonizer of poultry GIT is *Candidatus Arthromitus* (Danzeisen *et al.*, 2013; Gong *et al.*, 2007; Richards-Rios *et al.*, 2020) belonging to the family *Clostridiaceae* and the phylum Firmicutes. We found positive effects on several traits (Table 5), which is in agreement with other studies reporting positive correlations of this genus with animal performance traits (Danzeisen *et al.*, 2013; Johnson *et al.*, 2018). Both bacteria, *Bacillus* and some subspecies of *Enterococcus*, are considered as probiotic in chicken and Japanese quail (Cartman *et al.*, 2008; Hong *et al.*, 2005). *Bacillus* showed positive effects on several traits (Table 5). However, *Enterococcus* showed negative effects on FI and BWG, which may be due to the fact that *Enterococcus* is also known for pathogenesis and antibiotic resistance (Quednau *et al.*, 1998; Song *et al.*, 2019).

With regards to the trait microbial architecture it can tentative be concluded, that the traits are poly-microbial determined with some microbiota features exerting larger effects. In addition, the across-trait effects of the microbiota features point to substantial shared microbiota architecture for these traits. This is important for the development of hologenomic selection schemes that are targeting the host genome and the metagenome simultaneously (Weishaar *et al.*, 2020).

The models applied show strong similarities with corresponding genomic models. The genomic counterpart of model (1) is a model where the microbial relationship matrix *M* is replaced by a genomic relationship matrix built by dense SNP data (Yang *et al.*, 2011). The MWAS models (2) and (3) are closely related to genome-wide association studies (GWAS) frequently applied in livestock species, where single-marker as well as multi-marker models are used (reviewed in Gilbert *et al.*, 2016; Schmid and Bennewitz 2017). The strength of these association models is that nuisance factors can be included straightforwardly. In this study the random test-day effects and the random genetic animal effects (with the pedigree-based genetic relationship matrix) were included. Both explained significantly a part of the variance. The inclusion of a random genetic animal effect in GWAS models is important to model the population structure and we followed this in the MWAS model (2). Alternatively, the relationships of the animals could have been modeled by the M matrix. We tested this and found in general the same significant effects, although on a somewhat lower significance level (results not shown). The latter might result from the genus under consideration being included twice in the model, *i.e.*, as a fixed covariable and as random OTUs.

The applied models need large data sets. This is in contrast to so-called differential abundance analyses (Li 2015). These kind of studies are based on the comparison of the abundance of microbiota composition of previously selected groups of animals that differ with respect to their traits means. Naturally, also differential abundance analyses benefit from large data sets, but because group means are compared, they are applicable also to smaller data sets.

Conceptually, the main difference between the MWAS and the GWAS models is the use of relative abundances as regression variables instead of SNP genotypes. The relative abundances are compositional-type data with many zeros (Pawlowsky-Glahn *et al.*, 2015), which are multivariate with a unit sum. It is impossible to alter the relative abundance of one feature without altering at least one of the other abundances (reviewed in Li 2015). This limits the identification of causalities from MWAS results. Methods are available to handle microbiota compositional data (Shi *et al.*, 2016) and further research is needed to study the effect of incorporating these methods in the applied MWAS models. Thus, it is valid to conclude from the results of MWAS model (2) that the microbiota features are trait associated, but no inference of causality can be drawn. Since all features are considered simultaneously in equation (3), the problem is less evident for the results of the multi-OTU MWAS. Thus, this approach might serve as an ad hoc procedure to account for the compositional-type data structure. Further research is needed for the calculation of p values from the back-solved OTU effects as described for SNP effects obtained from genomic models by (Aguilar *et al.*, 2019).

The multi-OTU MWAS method treated the OTU as random with normally distributed homogeneous variances. These models are convenient to apply from a computational point of view, but the downside is that large OTU effects might be regressed back too strong and thus do not peak in the Manhattan plots. Alternative models allow for a heavy-tailed distribution of OTU effects (Maltecca *et al.*, 2019; Sanglard *et al.*, 2020).

## Conclusion

Except CaU, all traits were substantially influenced by the ileum microbiota composition and showed a substantial animal microbiota correlation. The latter points to the same microbiota features affecting multiple traits, which was confirmed by the results from the MWAS. The traits were poly-microbial in nature, with some microbiota features with large effects on the traits and many features with small or non-significant effects. The results might help to develop tailored breeding schemes that invoke microbial trait predictions. In this study ileum microbiota samples were used, but in practical breeding applications it is more convenient to use fecal samples. More research is needed to analyses if the microbiota composition in fecal samples are good quantitative trait predictors as well. They have to be confirmed in poultry species and lines such as laying hens or broiler chickens, which are economically more important than Japanese quail. The application of microbiome wide mixed linear models proved to be suitable to unravel the GIT microbial architecture of the traits, but have to be extended toward handling compositional type data.
